# Wheat Ammonium Transporter (AMT) Gene Family: Diversity and Possible Role in Host–Pathogen Interaction with Stem Rust

**DOI:** 10.3389/fpls.2017.01637

**Published:** 2017-09-20

**Authors:** Tianya Li, Kai Liao, Xiaofeng Xu, Yue Gao, Ziyuan Wang, Xiaofeng Zhu, Baolei Jia, Yuanhu Xuan

**Affiliations:** ^1^College of Plant Protection, Shenyang Agricultural University Shenyang, China; ^2^Department of Life Sciences, Chung-Ang University Seoul, South Korea

**Keywords:** TaAMT, ammonium, expressions, stem rust, wheat

## Abstract

Ammonium transporter (AMT) proteins have been reported in many plants, but no comprehensive analysis was performed in wheat. In this study, we identified 23 AMT members (hereafter TaAMTs) using a protein homology search in wheat genome. Tissue-specific expression analysis showed that *TaAMT1;1a, TaAMT1;1b*, and *TaAMT1;3a* were relatively more highly expressed in comparison with other *TaAMTs*. TaAMT1;1a, TaAMT1;1b, and TaAMT1;3a-GFP were localized in the plasma membrane in tobacco leaves, and *TaAMT1;1a, TaAMT1;1b*, and *TaAMT1;3a* successfully complemented a yeast 31019b strain in which ammonium uptake was deficient. In addition, the expression of *TaAMT1;1b* in an *Arabidopsis AMT* quadruple mutant (*qko*) successfully restored ${\mathrm{NH}}_{4}^{+}$ uptake ability. Resupply of ${\mathrm{NH}}_{4}^{+}$ rapidly increased cellular ${\mathrm{NH}}_{4}^{+}$ contents and suppressed expression of *TaAMT1;3a*, but not of *TaAMT;1;1a* and *TaAMT1;1b* expressions. Expression of *TaAMT1;1a, TaAMT1;1b*, and *TaAMT1;3a* was not changed in leaves after ${\mathrm{NH}}_{4}^{+}$ resupply. In contrast, nitrogen (N) deprivation induced *TaAMT1;1a, TaAMT1;1b*, and *TaAMT1;3a* gene expressions in the roots and leaves. Expression analysis in the leaves of the stem rust-susceptible wheat line “Little Club” and the rust-tolerant strain “Mini 2761” revealed that *TaAMT1;1a, TaAMT1;1b*, and *TaAMT1;3a* were specifically induced in the former but not in the latter. Rust-susceptible wheat plants grown under N-free conditions exhibited a lower disease index than plants grown with ${\mathrm{NH}}_{4}^{+}$ as the sole N source in the medium after infection with *Puccinia graminis* f. sp. *tritici*, suggesting that ${\mathrm{NH}}_{4}^{+}$ and its transport may facilitate the infection of wheat stem rust disease. Our findings may be important for understanding the potential function *TaAMTs* in wheat plants.

## Introduction

In most soils, nitrate (${\mathrm{NO}}_{3}^{-}$) and ammonium (${\mathrm{NH}}_{4}^{+}$) represent the major forms of nitrogen (N) uptake in higher plants. The ${\mathrm{NH}}_{4}^{+}$ ions accumulate in cells either by direct uptake from the rhizosphere via ammonium transporters (AMTs) or by reduction of ${\mathrm{NO}}_{3}^{-}$. The ${\mathrm{NH}}_{4}^{+}$ is then assimilated into glutamate via the glutamine synthetase/glutamate synthase (GS/GOGAT) cycle. Glutamine and asparagine have been identified as the major forms of organic N in the xylem and are translocated from the roots to the shoots (Fukumorita and Chino, 1982). The energy cost of reducing ${\mathrm{NO}}_{3}^{-}$ to ${\mathrm{NH}}_{4}^{+}$ involves the consumption of 12–26% of photosynthetically generated reductants. Therefore, the use of ${\mathrm{NH}}_{4}^{+}$ as an N source conserves a large amount of energy for plants (Bloom, 1997; Noctor and Foyer, 1998; Patterson et al., 2010). *AMT* genes have been identified in many plant species including *Arabidopsis thaliana* (Ninnemann et al., 1994; Gazzarrini et al., 1999; Sohlenkamp et al., 2000, 2002; Loqué and von Wirén, 2004; Yuan et al., 2007a, 2009), *Lycopersicon esculentum* (Lauter et al., 1996; Von Wirén et al., 2000), *Lotus japonicus* (Salvemini et al., 2001; Simon-Rosin et al., 2003; D’Apuzzo et al., 2004), *Brassica napus* (Pearson et al., 2002), *Oryza sativa* (Sonoda et al., 2003), *Zea mays* (Gu et al., 2013), and *Sorghum bicolor* (Koegel et al., 2013). In wheat, AMT *TaAMT1;1* was stimulated by an acidic pH *in vitro* (Sogaard et al., 2009). In addition, three *TaAMTs* identified their transcriptional regulation under arbuscular mycorrhizal (AM) fungi infection (Duan et al., 2015). However, no further information regarding wheat AMTs has been reported. Although ${\mathrm{NH}}_{4}^{+}$ is an energetically favorable N source, various plants exhibit toxic symptoms in response to high ${\mathrm{NH}}_{4}^{+}$ concentrations (Britto and Kronzucker, 2002).

In contrast to other grasses, rice is tolerant of ${\mathrm{NH}}_{4}^{+}$ and the tolerance relies on an energetically favorable equilibration of influx and efflux at elevated ${\mathrm{NH}}_{4}^{+}$ supplies (Britto et al., 2001). The uptake of high affinity ${\mathrm{NH}}_{4}^{+}$ into root cells is mediated by AMT type ${\mathrm{NH}}_{4}^{+}$ transporters, encompassing a family of 10 AMT paralogs that have been classified into four subfamilies in rice (Suenaga et al., 2003; Loqué and von Wirén, 2004; Ye et al., 2016). Among them, *AMT1;1, AMT1;2*, and *AMT1;3* are of particular importance; *AMT1;1* is constitutively expressed in the shoots and roots, while *AMT1;2* and *AMT1;3* are specifically expressed in the roots (Sonoda et al., 2003). *Arabidopsis* is a ${\mathrm{NH}}_{4}^{+}$-sensitive species, and all *AtAMT1* (*AtAMT1;1, AtAMT1;2*, and *AtAMT1;3*) and *AtAMT2;1* gene expression are suppressed at high ${\mathrm{NH}}_{4}^{+}$ concentrations (Sohlenkamp et al., 2000; Loqué et al., 2006). Furthermore, post-transcriptional and post-translational regulation in AMTs has been reported. In particular, the ${\mathrm{NH}}_{4}^{+}$-mediated phosphorylation at T460 in the cytosolic tail of AMT1;1 has been found to inhibit transporter activity in *A. thaliana* (Yuan et al., 2007b; Lanquar et al., 2009). In rice, the transcriptional regulation of *AMT* genes is dependent on the N nutritional status of the plant and on the external availability of different N forms. *AMT1;1* and *AMT1;2* are up-regulated in response to ${\mathrm{NH}}_{4}^{+}$; however, *AMT1;3* is up-regulated by N deprivation (Kumar et al., 2003; Sonoda et al., 2003). The overexpression of *AMT1;1* enhanced ${\mathrm{NH}}_{4}^{+}$ uptake and improved plant growth and yield production in rice, at least under specialized N fertilization conditions (Ranathunge et al., 2014). In contrast, the overexpression of *AMT1;3* resulted in poor growth and reduced ${\mathrm{NH}}_{4}^{+}$ uptake in rice (Bao et al., 2015). As rice plants use ${\mathrm{NH}}_{4}^{+}$ as a favorable N source, the importance of AMTs is evident, though their biological function remains unclear. The only information regarding the function of *AMT* genes is that ${\mathrm{NH}}_{4}^{+}$-triggered lateral root branching is controlled by *AMT1;3* in *Arabidopsis* (Lima et al., 2010).

Nitrogen status is closely associated with plant defense. In rice, treatment of the roots with glutamate induces systemic resistance to rice blast disease, partially through salicylic acid signaling (Kadotani et al., 2016). In *Arabidopsis, AMT1;1* alters basal defense, generating resistance against *Pseudomonas syringae* and *Plectosphaerella cucumerina* (Pastor et al., 2014), and the expressions of *AMT1;1, AMT1;2, AMT1;3*, and *AMT2;1* have been found to be altered by both biotic and abiotic stresses (Fagard et al., 2014). In sorghum, the expression of *SbAMT3;1* and *SbAMT4* was greatly induced locally in roots colonized by AM fungi (Koegel et al., 2013). Wheat stem rust is one of the most serious diseases of wheat worldwide (Pardey et al., 2013). In China, it has been effectively controlled through the development of resistant cultivars and effective resistant genes, particularly the 1B/1R translocation gene *Sr31*, in different epidemiological regions since the 1970s (Cao and Chen, 2010). However, a new strain of stem rust pathogen designated as Ug99 (TTKS), expressing virulence to *Sr31*, was first identified in Uganda in 1998 (Pretorius et al., 2000). It has since spread throughout the major wheat growing regions of the world such as Ethiopia, Zimbabwe, Mozambique, Kenya, Sudan, Yemen, and Tanzania (Singh et al., 2008). Ug99 and related strains threaten global wheat production because they are virulent on widely used cultivars that had otherwise been effective for many years (Yu et al., 2010). This has initiated renewed genetic research into wheat to identify tolerant strains and the regulatory mechanism thereof.

In the present study, we identified and characterized *TaAMTs* in wheat plants. The homology of 23 *TaAMTs* was compared with *AMTs* from other species and their tissue-specific or ${\mathrm{NH}}_{4}^{+}$-mediated expressions were analyzed. In addition, the localization of TaAMT1;1 proteins and their functions were analyzed using a yeast ${\mathrm{NH}}_{4}^{+}$ uptake deficient strain and an *Arabidopsis* AMT *qko* mutant. *TaAMT1;1* expression in wheat was also examined upon *Puccinia graminis* f. sp. *tritici* (*Pgt*) infection.

## Materials and Methods

### Plant Growth

*Arabidopsis* seeds (*qko, qko+AtAMT1;1*, and *qko+TaAMT1;1b*) (Yuan et al., 2007a) were surface sterilized and kept in a 4°C chamber for 2 days. The seeds were planted in modified 0.5× MS medium containing 1 mM KNO_3_ as the sole N source. *Arabidopsis* were cultured in the chamber at 22°C with 12 h/12 h: light/dark cycle. Three-day-old plants were transferred to the same medium containing 0 or 10 mM methyl-ammonium (MeA) and grown for another 4 days. In order to analyze the cellular ${\mathrm{NH}}_{4}^{+}$ contents, the *Arabidopsis* mutants (*qko, qko+AtAMT1;1*, and *qko+TaAMT1;1b*) were planted in 0.5× MS medium and grown for 7 days, after which their complete root systems from 30 plants were collected.

The stem rust-susceptible wheat (*Triticum aestivum*) line “Little Club” (LC) was used in the experiments to examine the effects of ${\mathrm{NH}}_{4}^{+}$ on *TaAMT1* gene expression. Germinated seeds were grown in deionized water in a greenhouse for 2 weeks to consume all the nutrient solution in the endosperm. Wheat plants were cultured in the chamber at 21°C with 12/12: light/dark cycle. The seedlings were then grown for another 3 days in N-free nutrient solution (–N basal salt: 7 μM Na_2_HPO_4_, 16 μM KCl, 7 μM CaCl_2_.2H_2_O, 15 μM MgCl_2_.6H_2_O, 36 μM FeSO_4_.7H_2_O, 9 μM MnSO_4_.4H_2_O, 45 μM H_3_BO_4_, 3 μM ZnSO_4_.7H_2_O, 0.2 μM CuSO_4_.7H_2_O, 0.05 μM Na_2_MoO_4_.2H_2_O) (Abiko et al., 2005), after which they were transferred to a nutrient solution containing 0.5 mM (NH_4_)_2_SO_4_ at pH 5.5. Whole roots and leaves were harvested at 0, 1, 3, and 6 h following the provision of 0.5 mM (NH_4_)_2_SO_4_. Two-week-old LC plants grown in water were transferred to a nutrient solution (–N basal salt) containing 0.5 mM (NH_4_)_2_SO_4_. After 3 days of growth, the plants were transferred to the same nutrient solution without the N source (–N basal salt). Whole roots and leaves were collected after 0, 1, 2, and 3 days of N deprivation. Seventeen-day-old LC plant roots and leaves, as well as 2-month-old plant stems and flowers, were harvested for RNA extraction.

### Stem Rust Infection

For inoculation of the urediniospores of *Pgt* (race 21C3CTHTM), the urediniospores were separately inoculated to stem rust-susceptible wheat (*T. aestivum*) “LC” and stem rust resistant line “Mini 2761” seedlings once the primary leaves had fully expanded while the secondary leaves were being sprouted. The urediniospores of *Pgt* (race 21C3CTHTM) were propagated by inoculation of LC leaves in the growth chamber. The inoculated seedlings were kept in moist conditions for 14–16 h, and then cultured at 21 ± 1°C, 12 h/12 h: light/dark cycle and a light intensity of 5.8–6.0 klx. The infection types were recorded according to six classes of standards (Supplementary Figure S1; Roelfs and Martens, 1988). To analyze N fertilization dependent stem rust disease index, seeds were grown in deionized water for 2 weeks before being transferred to –N basal salt containing 0.5 mM (NH_4_)_2_SO_4_ solution (pH 5.5) for another 2 weeks prior to inoculation of *Pgt*.

### Molecular Phylogenetic Analysis Using Maximum Likelihood

To search AMT amino sequences in wheat, the rice, *Arabidopsis*, and potato AMT sequences were used as a bait and searched in the Uniprot database^1^ using BLAST with *e*-value cutoff of *e*-10 (Pearson, 2013). The retrieved sequences are listed in Supplementary Table S1. The multiple sequence alignments were performed by ClustalW and the evolutionary history was inferred using maximum likelihood based on the JTT matrix-based model. The tree with the highest log likelihood (-12,240.60) is built. Initial tree(s) for the heuristic search were obtained automatically by applying the Neighbor-Join and BioNJ algorithms to a matrix of pairwise distances estimated using a JTT model. The topology with the superior log likelihood value was then selected. The analysis involved 42 amino acid sequences. All positions containing gaps and missing data were eliminated. There was a total of 49 positions in the final dataset. Evolutionary analyses were conducted in MEGA7 (Kumar et al., 2016).

### RNA Extraction and qRT-PCR

Total cellular RNA was isolated from 20 plant tissues with TRIzol (Takara, Dalian, Liaoning, China), and 2 μg of total RNA was subsequently treated with RQ1 RNase free DNase (Promega, Madison, WI, United States) to eliminate genomic DNA contamination. For cDNA synthesis, a GoScript Reverse Transcription Kit was used following the manufacturer’s instructions (Promega, Madison, WI, United States). Subsequently, qRT-PCR was performed in triplicate using the SYBR Green Mix (Bio-Rad). The three replicates of PCR in each time were analyzed for one sample and the experiments were repeated at least three times. The PCR products were quantified using the Illumina Research Quantity software Illumina Eco 3.0 (Illumina, San Diego, CA, United States). The values of each sample were first normalized against *TaEF1α* levels from the same samples, and next compared with indicated control group value to analyze the ratio for each gene. Changes in gene expression were calculated using the 2^-ΔΔC_T_^ method (Han et al., 2006). The primers used for qRT-PCR are listed in Supplementary Table S2.

### Yeast Complementation Assay

The ammonium uptake deficient yeast strain 31019b (*Δmep1, Δmep2, Δmep3, ura3*; Marini et al., 1997) was obtained from the Frommer Laboratory (Carnegie Institution for Science). A pDRf1-GW (Xuan et al., 2013) vector harboring *TaAMT1;1a, TaAMT1;1b*, or *TaAMT1;3a* was transformed into the yeast cells. The successful transformants were screened by growing of yeast cells in the SD/-ura solid medium. Each transformant was plated on yeast N base media containing 1 mM NH_4_Cl or 1 mM arginine, and yeast growth was monitored at 28°C for 3 days. The primers used for cloning the *TaAMT* genes are listed in Supplementary Table S1.

### *Arabidopsis qko* Mutant Complementation

Nucleotides of *TaAMT1;1b* ORF were fused to 1.5 kb of the *AtAMT1;1* promoter sequence and cloned into the pABind vector (Bleckmann et al., 2010). The pABind-*pAtAMT1;1*–*TaAMT1;1b* construct was transformed into the *Arabidopsis qko* mutant background via *Agrobacterium*-mediated transformation. *qko* mutant has mixed Col-0 and Ws-2 genomes; therefore, the *qko+AtAMT1;1* was used as an control. The transgenic plant seeds were selected in the plates containing hygromycin, and around 20 individual plants were selected. For analyzing MeA uptake and cellular ${\mathrm{NH}}_{4}^{+}$ contents, three independent lines were further examined.

### Localization of *TaAMT1;1* in Plants

Nucleotides of *TaAMT1;1* ORFs were cloned into a pABindGFP (35S promoter) destination plasmid (Bleckmann et al., 2010) followed by transient expression in *Nicotiana benthamiana* leaves using the *Agrobacterium*-mediated transient expression method (Kim et al., 2009). Green fluorescent protein fluorescence was detected under a confocal microscope (SP5; Leica).

### Determination of Ammonium Contents

Enzymatic determination of the ammonium contents in roots was performed using an F-Kit (Roche) according to the manufacturer’s instructions (Oliveira et al., 2002).

### Statistical Analysis

Statistical calculations were conducted using Prism 5 (GraphPad, San Diego, CA, United States). Significant differences between two groups were analyzed by Student’s *t*-test. Comparisons between more than two groups were performed by using one-way ANOVA followed by Bonferroni’s multiple comparison test. *P*-values of <0.05 were considered statistically significant.

## Results

### Identification of TaAMTs and Phylogenetic Relationships between AMTs

**FIGURE 1:**
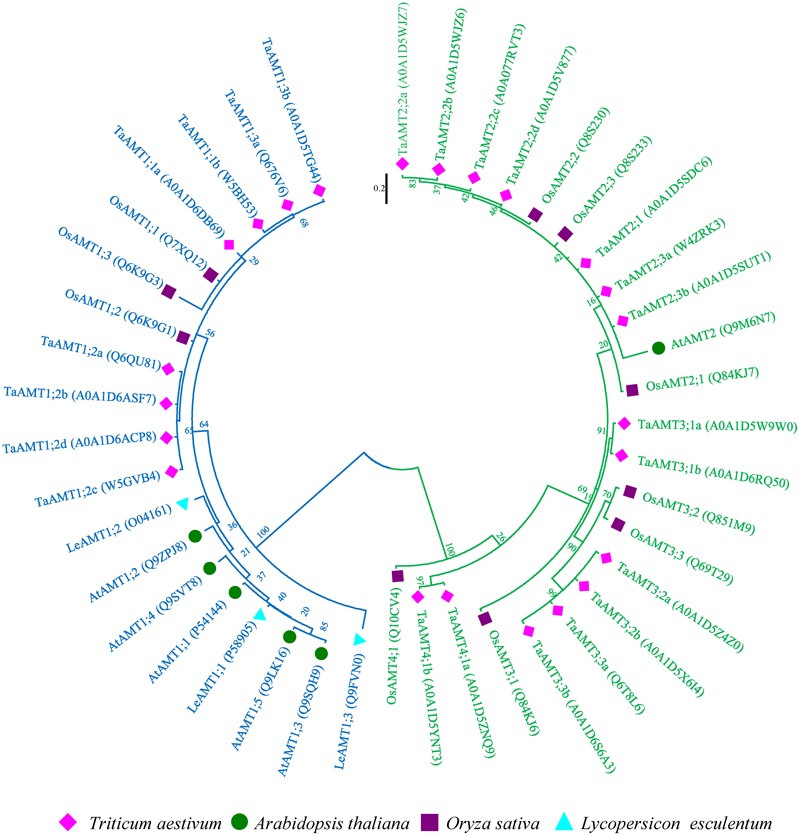
Phylogenetic tree of AMTs from *Oryza sativa, Arabidopsis thaliana, Triticum aestivum*, and *Solanum lycopersicum.* The phylogenetic tree was constructed using the maximum-likelihood method. The unrooted tree was generated using ClustalW in MEGA 7.0 using AMT amino acid sequences from *O. sativa* (OsAMTs; dark blue color), *A. thaliana* (AtAMTs; dark orange color), *T. aestivum* (TaAMTs; light green color), and *S. lycopersicum* (LeAMTs; light blue color). AMT1s are marked with blue and others are marked with green.

High affinity ${\mathrm{NH}}_{4}^{+}$ transport is mediated by AMT family transporters. To isolate wheat TaAMTs sequences, 10 rice, 6 *Arabidopsis*, and 3 potato AMT sequences were used as baits to obtain the amino sequences of AMTs in wheat. A total of 23 AMTs (among 25 identified in Uniprot database, 2 *AMTs* were duplicated) were identified in *T. aestivum*, and were designated as TaAMTs on the basis of their similarity to OsAMTs, AtAMTs, and LeAMTs (**Figure 1** and Supplementary Table S1). In order to understand the evolutionary relationships between TaAMTs and AMTs from other plant species, the amino acid sequences of 10 AMTs from *O. sativa*, 6 AMTs from *A. thaliana*, and 3 AMTs from *Solanum lycopersicum* were collected and aligned. An unrooted phylogenetic ML tree was constructed and the results indicated that the AMTs from four species could be classified into four sub-groups (AMT1, AMT2, AMT3, and AMT4). Among these, all the AMT1 proteins clustered together with high bootstrap support (100%), while AMT2, AMT3, and AMT4 were closely associated in the phylogenetic tree. Furthermore, the tree topology was independent of the methods used for the phylogenetic reconstruction (data not shown). Rice and wheat were clustered more closely, whereas the tomato and *Arabidopsis* AMTs were clustered relatively distantly (**Figure 1**).

### Tissue-Specific Expression of TaAMT Genes

**FIGURE 2:**
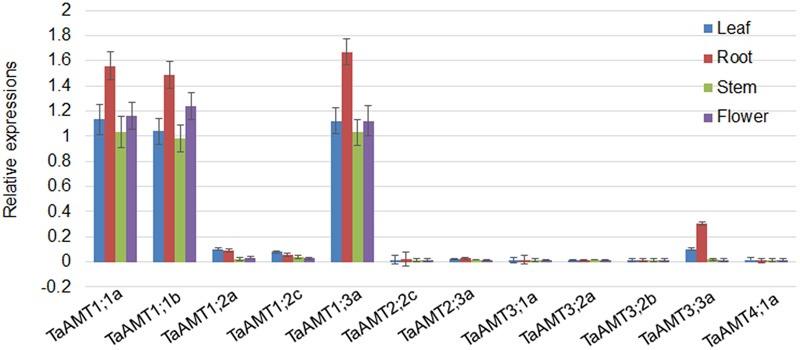
Tissue-specific expression of *TaAMT* genes in wheat. Expression levels of *TaAMTs* in the leaves, roots, stems, and flowers were analyzed. The expression patterns were analyzed by qRT-PCR. The *TaEF1α* gene was used as the internal control. The experiments were repeated three times.

To examine the expression patterns of *TaAMT* genes, the roots, leaves, stems, and flowers were collected for RNA extraction. qRT-PCR was performed to analyze 12 TaAMT gene expressions among the 23 *TaAMT* members, and the results showed that two *TaAMT1;1* group genes (*1;1a* and *1;1b*) and *TaAMT1;3a* were highly expressed, while the remainder of the *TaAMT* genes were barely detected (**Figure 2**). The *TaAMT1;1* group genes (*1;1a* and *1;1b*) and *TaAMT1;3a* exhibited similar patterns and showed the highest expression levels in the roots, while similar expression levels were found in the leaves, stems, and flower tissues. The expression levels of *TaAMT1;2a* and *TaAMT1;2c* were relatively higher in the leaves and roots than in the stems and flowers. The expression levels of the *TaAMT3;2* group genes were not as high as the *TaAMT1;1* group genes, and *TaAMT3;3a* exhibited the highest expression levels in the roots, and the lowest in the stems and flowers. The remaining genes either showed reduced expression levels or were not detected (**Figure 2**).

### Two TaAMT1;1 and TaATM1;3a Proteins Localize at the Plasma Membrane and Transport ${\mathrm{NH}}_{4}^{+}$ in Yeast

**FIGURE 3:**
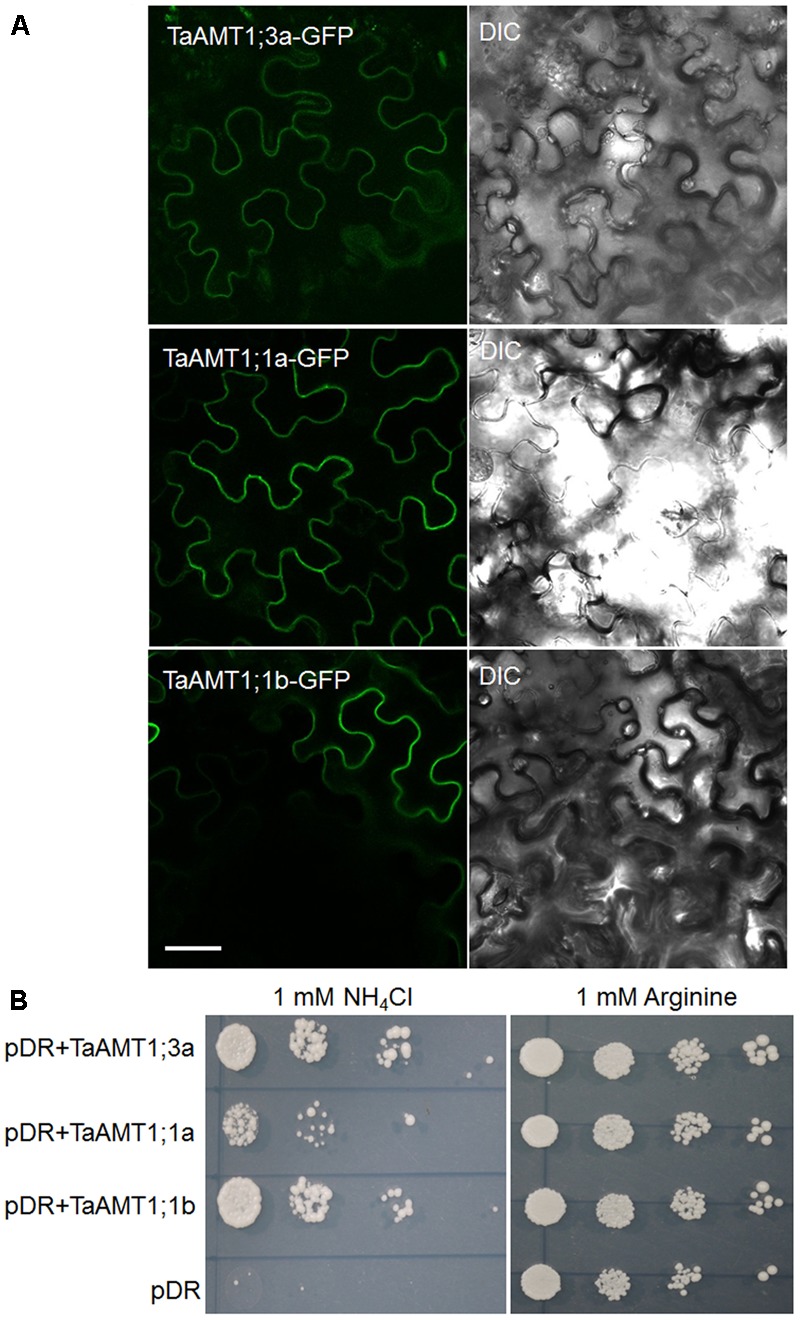
Localization and functional analysis of TaAMT1;1a, TaAMT1;1b, and TaAMT1;3a proteins. *Agrobacterium*-mediated transient expression of TaAMT1;1a, TaAMT1;1b, and TaAMT1;3a-GFP constructs in *N. benthamiana* leaves. GFP fluorescence and bright field images are shown in the left and right panels, respectively. Bar = 20 μm **(A)**. The functions of TaAMT1;1a, TaAMT1;1b, and TaAMT1;3a genes were analyzed by complementation of an ${\mathrm{NH}}_{4}^{+}$ uptake defective yeast strain 31019b (*Δmep1, Δmep2, Δmep3, ura3*). Yeast cells were transformed expressing TaAMT1;1a, TaAMT1;1b, and TaAMT1;3a via a pDRf1 (pDR) vector or an empty vector, and were tested for growth complementation on yeast N base plates supplemented with 1 mM NH_4_Cl or 1 mM arginine (Arg). The empty vector (pDRf1) was used as the control. The yeast cells were grown at 30°C for 3 days **(B)**.

Since *TaAMT1;1* group genes (*1;1a* and *1;1b*) and *TaAMT1;3a* were the dominant TaAMT members, the subcellular localization of TaAMT1;1a, TaAMT1;1b, and TaAMT1;3a was monitored. *GFP* coding sequences were C-terminally fused to *TaAMT1;1a, TaAMT1;1b*, and *TaAMT1;3a* and the fusion proteins were transiently expressed in *N. benthamiana* leaves via *Agrobacterium*-mediated transformation. Two days after infection, the TaAMT-GFP signal was detected on the plasma membrane (**Figure 3A**). TaAMTs conserved with other characterized AMTs from different plant species in a phylogenetic tree (**Figure 1**). Therefore, we examined their ${\mathrm{NH}}_{4}^{+}$ transport activity. Since the *TaAMT1;1* group of genes and *TaAMT1;3a* were most strongly expressed in all the tissues tested, the transport activity of these three proteins was analyzed (**Figure 2**). We used the yeast mutant complement approach to test the transport activity of TaAMT1;1a, TaAMT1;1b, and TaAMT1;3a when ${\mathrm{NH}}_{4}^{+}$ was supplied. Coding sequences of *TaAMT1;1a, TaAMT1;1b*, and *TaAMT1;3a* were cloned into the yeast expression vector pDRf1-GW, and the three genes were expressed in the yeast strain 31019b (*Δmep1, Δmep2, Δmep3, ura3*), which is deficient in ${\mathrm{NH}}_{4}^{+}$ uptake (Marini et al., 1997). Yeast cell growth was monitored in the media containing 1 mM NH_4_Cl or 1 mM arginine. The results indicate that TaAMT1;1a, TaAMT1;1b, and TaAMT1;3a are able to transport ${\mathrm{NH}}_{4}^{+}$ into yeast cells (**Figure 3B**).

### Complementation of the *qko* Mutant by Heterologous Expression of *TaAMT1;1b*

**FIGURE 4:**
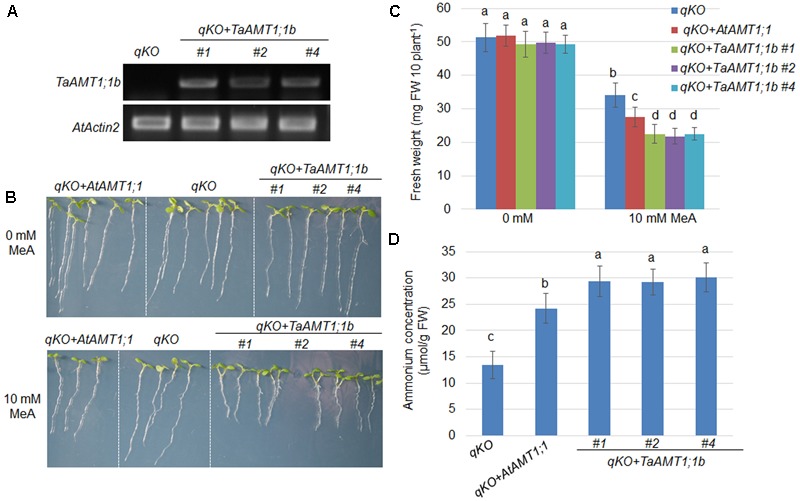
Complementation of the *Arabidopsis* ${\mathrm{NH}}_{4}^{+}$ uptake defective mutant *qko* by *TaAMT1;1b*. *TaATM1;1b* was expressed in the *qko* background under the control of the *AtAMT1;1* promoter. Expression levels of *TaATM1;1b* were monitored in the *TaATM1;1b* transgenic plant roots (*#1, #2*, and *#4*), and *AtActin2* was used as a loading control **(A)**. Methyl-ammonium (MeA) dependent growth of *qko, qko+AtAMT1;1*, and *qko+TaAMT1;1b* was photographed. Three-day-old seedlings grown in 1 mM KNO_3_ containing modified 0.5× MS medium were transferred to the same medium containing either 0 or 10 mM MeA. Plant growth was photographed after 4 days **(B)**. Total fresh weight of the seedlings (30 seedling for each line) shown in **(B)** was measured. Significant differences between *qko* and *qko+AtAMT1;1* or *qko+TaAMT1;1b* were assessed **(C)**. ${\mathrm{NH}}_{4}^{+}$ contents from the roots of 7-day-old *qko, qko+AtAMT1;1*, and *qko+TaAMT1;1b* plants grown in 0.5× MS medium were measured **(D)**. Significant differences at *P* < 0.05 level are indicated by different letters.

Two TaAMT1;1 genes (*TaAMT1;1a* and *TaAMT1;1b*) are able to complement the yeast mutant 31019b in which ${\mathrm{NH}}_{4}^{+}$ uptake is deficient, and TaAMT1;1b showed higher affinity than TaATM1;1a in transport of ${\mathrm{NH}}_{4}^{+}$ (**Figure 3B**). Therefore, *TaAMT1;1b* function was further analyzed in plants. The *Arabidopsis* quadruple mutant (*qko*) missing *AMT1;1, AMT1;2, AMT1;3*, and *AMT2;1* greatly reduced ${\mathrm{NH}}_{4}^{+}$ uptake ability (Yuan et al., 2007a). Since gene transformation in wheat is challenging, *TaAMT1;1b* was selected and expressed in *qko* under the control of a 1.5 kb *AtAMT1;1* promoter. The RT-PCR results indicate that *TaAMT1;1b* was expressed in the three independent transgenic *Arabidopsis* lines (*#1, #2*, and *#4*), while no visible transcript of *TaAMT1;1b* was detected in the *qko* mutant (**Figure 4A**). MeA uptake was tested in *qko, qko+AtAMT1;1* (in which *AtAMT1;1* was expressed by its own promoter), and three independent *qko+TaAMT1;1b* plants. Three-day-old seedlings grown on modified 0.5× MS medium containing 1 mM KNO_3_ as the sole N source were transferred to the same medium with or without 10 mM MeA. *qko, qko+AtAMT1;1*, and *qko+TaAMT1;1b* exhibited similar growth patterns without the addition of MeA. However, the growth of *qko+AtAMT1;1* and *qko+TaAMT1;1b*, but not *gko*, was severely affected after the addition of MeA to the growth medium (**Figure 4B**). Root growth and seedling fresh weight of *qko+TaAMT1;1b* plants was more severely affected by MeA than *qko+AtAMT1;1* plants (**Figures 4B,C**). Additionally, cellular ${\mathrm{NH}}_{4}^{+}$ contents were measured from the 7-day-old *qko, qko+AtAMT1;1*, and *qko+TaAMT1;1b* plant roots, and the results indicated that the roots of *qko+AtAMT1;1* and *qko+TaAMT1;1b* contained more ${\mathrm{NH}}_{4}^{+}$ than the roots of *qko* (**Figure 4D**).

### N Dependent Expressions of *TaAMT1;1* Genes

**FIGURE 5:**
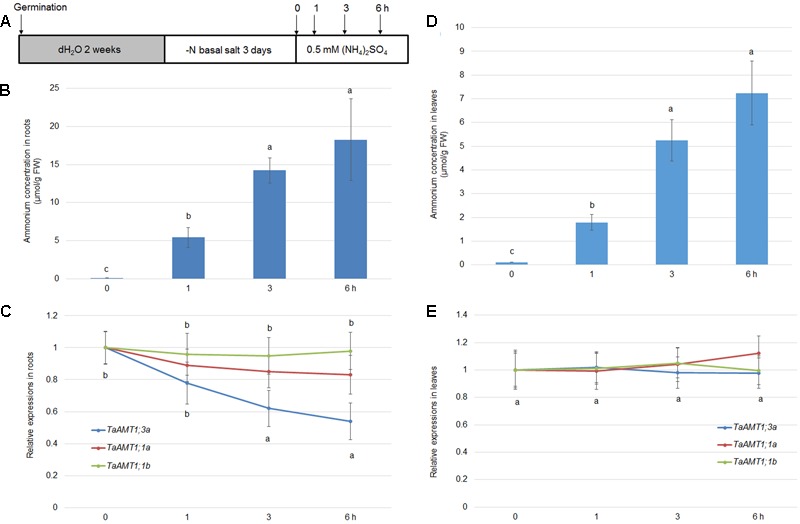
${\mathrm{NH}}_{4}^{+}$-dependent transcriptional changes in *TaAMT1;1* genes. Seventeen-day-old seedlings grown as described in the section “Materials and Methods” were transferred to 0.5 mM (NH_4_)_2_SO_4_ containing solution (pH 5.5). Total roots and leaves of 17-day-old plants were sampled at 0, 1, 3, and 6 h after the addition of ${\mathrm{NH}}_{4}^{+}$ **(A)**. Endogenous ${\mathrm{NH}}_{4}^{+}$ levels measured in the roots of 17-day-old plants (*n* > 10). Data represent means ± SE (*n* = 3) **(B)**. qRT-PCR was performed to determine the expression levels of *TaAMT* genes in the roots. The level of *TaAMT1;1* genes before ${\mathrm{NH}}_{4}^{+}$ treatment was defined as 1. The relative ratio shown is the ratio against 1 **(C)**. Endogenous ${\mathrm{NH}}_{4}^{+}$ levels measured in the leaves of 17-day-old plants. Data represent means ± SE (*n* = 3) **(D)**. qRT-PCR was performed to determine the expression levels of *TaAMT* genes in the leaves **(E)**. The levels of *TaAMT1;1* genes before ${\mathrm{NH}}_{4}^{+}$ treatment was defined as 1. The relative ratio shown is the ratio against 1. qRT-PCR was performed to determine the expression levels of *TaAMT1* genes in the roots. The levels of *TaAMT1;1* genes before N deprivation were defined as 1. The relative ratio shown is the ratio against 1. Significant differences at *P* < 0.05 level are indicated by different letters.

The expression of *AMT* genes is sensitive to exogenous N conditions. In *Arabidopsis*, the *AMT1* genes were repressed upon the resupply of ${\mathrm{NH}}_{4}^{+}$, while the *AMT1;1* and *AMT;12* genes in rice were highly induced by ${\mathrm{NH}}_{4}^{+}$ application (Sonoda et al., 2003; Loqué et al., 2006). To examine the ${\mathrm{NH}}_{4}^{+}$-mediated expression of *TaAMT1;1a, TaAMT1;1b*, and *TaAMT1;3a*, 17-day-old wheat seedlings grown under N-free conditions were treated with 1 mM ${\mathrm{NH}}_{4}^{+}$ for 0, 1, 3, and 6 h, and their whole roots and leaves were sampled (**Figure 5A**). Prior to analyzing the ${\mathrm{NH}}_{4}^{+}$-dependent expression patterns of *TaAMT1;1a, TaAMT1;1b*, and *TaAMT1;3a*, the cellular ${\mathrm{NH}}_{4}^{+}$ contents were measured in the roots. After transferring the wheat plants from the N-free medium to the solution containing ${\mathrm{NH}}_{4}^{+}$, the ${\mathrm{NH}}_{4}^{+}$ contents were rapidly increased up to 6 h of treatment (**Figure 5B**). qRT-PCR results showed that *TaAMT1;3a* was suppressed while *TaAMT1;1a* and *TaAMT1;1b* were not suppressed by ${\mathrm{NH}}_{4}^{+}$ treatment in the roots (**Figure 5C**). The expression levels of the remaining *TaAMT* members were not altered by ${\mathrm{NH}}_{4}^{+}$ (data not shown). Furthermore, the ${\mathrm{NH}}_{4}^{+}$ contents in the leaves were monitored before and after ${\mathrm{NH}}_{4}^{+}$ treatment. The ${\mathrm{NH}}_{4}^{+}$ levels increased and reached a maximum at 6 h of treatment, but the contents were much lower than in the roots (**Figure 5D**). However, the ${\mathrm{NH}}_{4}^{+}$-mediated expression levels of *TaAMT1;1a, TaAMT1;1b*, and *TaAMT1;3a* were unaltered in the leaves (**Figure 5E**).

**FIGURE 6:**
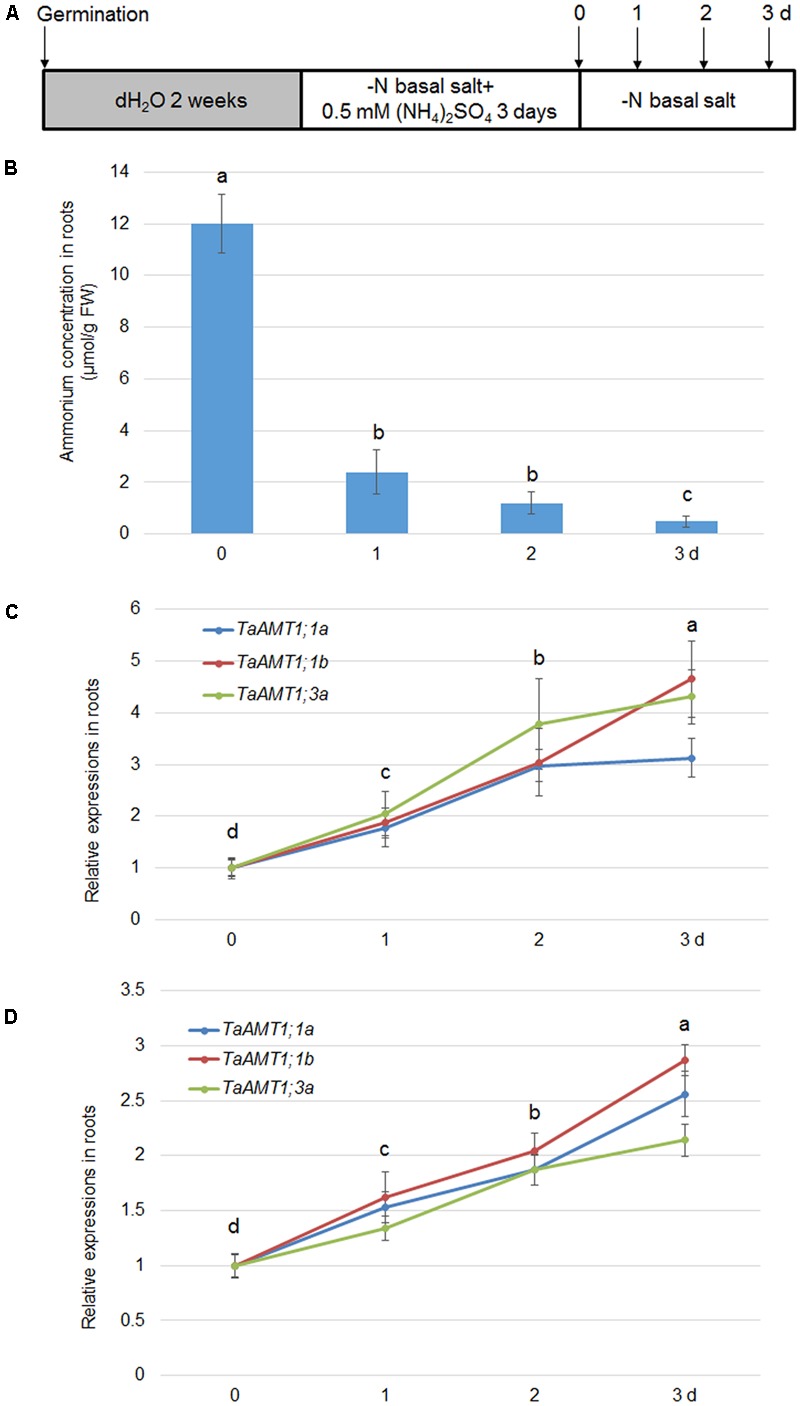
Effect of N-deprivation on *TaAMT1;1* gene expressions. For testing N-deprivation dependent gene expressions, the plants were grown as described in the section “Materials and Methods” **(A)**. Endogenous ${\mathrm{NH}}_{4}^{+}$ levels measured in the roots of 17-day-old plants. Data represent means ± SE (*n* = 3) **(B)**. The N-deficiency dependent expression of *TaAMT1;1a, TaAMT1;1b*, and *TaAMT1;3a* were analyzed in the roots **(C)** and leave **(D)** samples. Significant differences at *P* < 0.05 level are indicated by different letters.

*AMT* expression is not only sensitive to ${\mathrm{NH}}_{4}^{+}$ conditions, but also responds to N deprivation in *Arabidopsis* and rice (Sonoda et al., 2003; Loqué et al., 2006). Therefore, expression of *TaAMT1;1a, TaAMT1;1b*, and *TaAMT1;3a* was analyzed upon N starvation. The 14-day-old seedlings grown in water were grown in nutrient solution containing ${\mathrm{NH}}_{4}^{+}$ for another 3 days. They were subsequently transferred to an N deprived solution (**Figure 6A**). After the transfer, the cellular ${\mathrm{NH}}_{4}^{+}$ levels rapidly decreased in the roots, and the lowest level was observed after 3 days (**Figure 6B**). Simultaneously, qRT-PCR was used to determine the N-status-dependent expressions of *TaAMT1;1a, TaAMT1;1b*, and *TaAMT1;3a*. The results indicated that *TaAMT1;1a, TaAMT1;1b*, and *TaAMT1;3a* tested were induced under N starved conditions. The *TaAMT1;1a, TaAMT1;1b*, and *TaAMT1;3a* were highly induced, especially on the third day in the roots (**Figure 6C**), and leaves (**Figure 6D**).

### *TaAMT1;1* and *TaAMT1;3a* Expressions Are Induced by *Pgt* Infection

**FIGURE 7:**
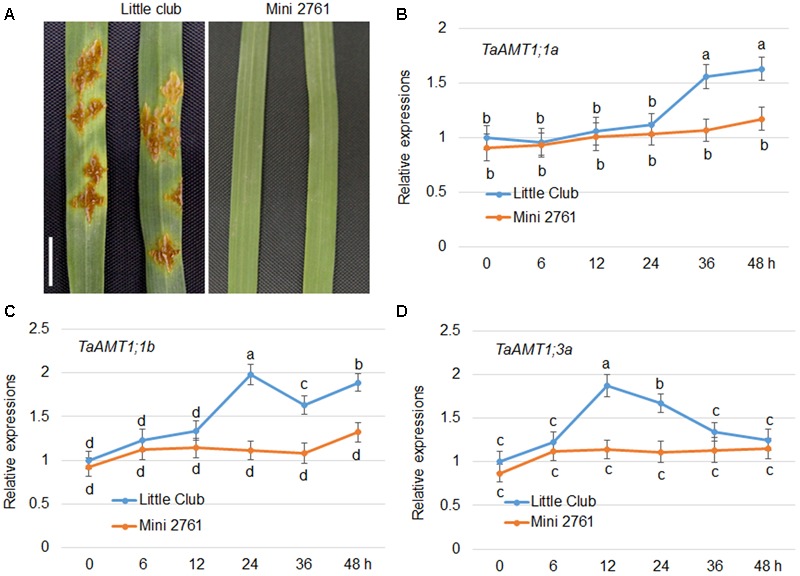
Urediniospore infection-mediated expression patterns of *TaAMT1;1* genes. Phenotype of uredinia growth in two different wheat varieties. Leaves of 4-week-old LC and Mini 2761, a stem rust susceptible and immune line, respectively, were inoculated with urediniospores. After 7 days of infection, the uredinia on the surface of the leaves were photographed. Bar = 1 cm. Leaves of 4-week-old LC and Mini 2761 were inoculated with urediniospores, and the leaves were collected for RNA extraction 0, 6, 12, 24, 36, and 48 h after inoculation. *TaAMT1;1* gene expressions were monitored by qRT-PCR **(A)**. The levels of *TaAMT1;1a* **(B)**, *TaAMT1;1b* **(C)**, and *TaAMT1;3a* **(D)**, before inoculation (0 h) was defined as 1. The relative ratio shown is the ratio against 1. The experiments were repeated three times and significant differences at *P* < 0.05 level are indicated by different letters.

*AtAMT1;1* has been shown to alter basal defense against *P. syringae* and *P. cucumerina* (Pastor et al., 2014). In wheat, three *TaAMTs* levels were altered by AM fungi infection (Duan et al., 2015). To dissect whether there is a relationship between *Pgt* infection and expression of *TaAMT1;1* genes, the stem rust-susceptible line LC (Roelfs and Martens, 1988) and resistant line Mini 2761 (Luan et al., 2013) were utilized, respectively. Following inoculation, urediniospore multiplication was detected in the leaves of the LC plants, while Mini 2761 exhibited lack of lesions in leaves (**Figure 7A**). Furthermore, the urediniospore inoculation-induced expression of *TaAMT1;1a, TaAMT1;1b*, and *TaAMT1;3a* genes was examined. The expression levels of *TaAMT1;1a, TaAMT1;1b*, and *TaAMT1;3a* differed insignificantly between the leaves of LC and Mini 2761. *TaAMT1;3a* was induced at 12 and 24 h (**Figure 7B**); *TaAMT1;1a* was induced at 36 and 48 h (**Figure 7C**); and *TaAMT1;1b* was induced at 24, 36, and 48 h after inoculation in LC only (**Figure 7D**). The expression levels of the three genes showed trifle difference following inoculation of the leaves of Mini 2761.

**FIGURE 8:**
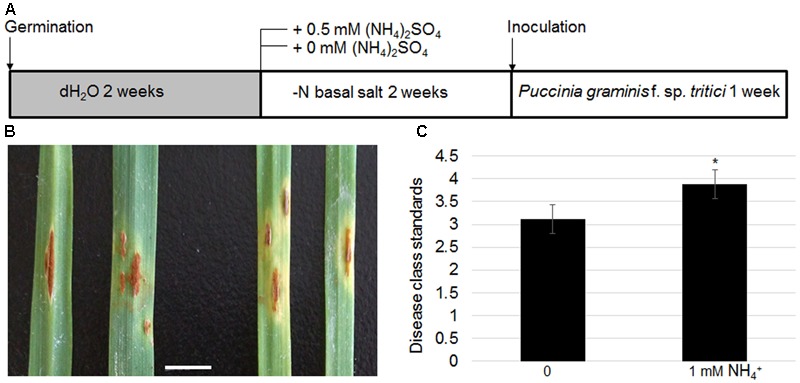
Effects of N-deficient growth conditions on wheat stem rust disease. Schematic diagram showing plant growth conditions for testing N effects on stem rust disease. Four-week-old LC seedling was inoculated with *Pgt* **(A)**. Photograph of uredinia growth in the leaves of LC grown on ${\mathrm{NH}}_{4}^{+}$-supplied (left panel) or N-free media (right panel). Bar = 1 cm **(B)**. Disease class standards were calculated following the international standard for wheat stem rust disease corresponding to the phenotypes shown in **(B,C)**. Significant differences were analyzed (^∗^*P* < 0.05).

As *TaAMT* genes were specifically induced in a stem rust-susceptible line (LC) after inoculation of the urediniospores, the effects of N availability in the growth medium on wheat stem rust disease were examined further. The LC plants were grown without any nutrient supply for the first 2 weeks after germination, following which they were transferred to a medium containing either 0 or 1 mM ${\mathrm{NH}}_{4}^{+}$. After 2 weeks of growth, the urediniospores were inoculated evenly on the surface of the leaves (**Figure 8A**). The symptoms were monitored after another 2 weeks of inoculation. The results indicated that the plants grown under N-free conditions exhibited lighter colored uredinia as well as chlorosis surrounding the uredinium-formed regions (**Figure 8B**). Disease class standards were calculated for the symptoms shown in **Figure 7B** following international wheat rust disease standards (Abiko et al., 2005). The results indicate that plants grown on the medium containing ${\mathrm{NH}}_{4}^{+}$ were on average Class 4, while the plants grown under N-free conditions were on average Class 3 (**Figure 8C**). These results suggest that N deficiency inhibits stem rust disease in wheat.

## Discussion

Nitrogen is an important nutrient for plant growth and production, accounting for 2% of the dry weight. Ammonium is the common N source for higher plants, and acquisition of ${\mathrm{NH}}_{4}^{+}$ from the rhizosphere occurs through ${\mathrm{NH}}_{4}^{+}$ transporters (AMTs) in plants. These AMTs have been characterized in many plant species including *Arabidopsis*, rice, tomato, rape, maize, and sorghum (Ninnemann et al., 1994; Lauter et al., 1996; Gazzarrini et al., 1999; Sohlenkamp et al., 2000, 2002; Von Wirén et al., 2000; Salvemini et al., 2001; Pearson et al., 2002; Simon-Rosin et al., 2003; Sonoda et al., 2003; Suenaga et al., 2003; D’Apuzzo et al., 2004; Loqué and von Wirén, 2004; Yuan et al., 2007a,b, 2009; Gu et al., 2013; Koegel et al., 2013; Ye et al., 2016). In this study, we identified AMT members in a major global crop, wheat.

Based on the sequence similarity search, 23 AMTs were retrieved from the *T. aestivum* genome. In phylogenetic results showed that four AMT group (AMT1, AMT2, AMT3, and AMT4) members were all present in the wheat genome (**Figure 1**). In *Arabidopsis* and rice, the AMT1 group genes are expressed either ubiquitously or tissue specifically (Sonoda et al., 2003; Loqué et al., 2006). In rice, the diverse genomic structure and transmembrane helices numbers of *OsAMT* members have been identified (Ye et al., 2016). We found that *TaAMT1;1a, TaAMT1;1b*, and *TaAMT1;3a* were strongly expressed, while the other *TaAMT* members were weakly expressed or otherwise not detected in wheat (**Figure 2**). To analyze the localization of TaAMT1;1a, TaAMT1;1b, and TaAMT1;3a, GFP fusion proteins were expressed in *N. benthamiana* leaves. Observation of GFP signal indicated that TaAMT1;1a, TaAMT1;1b, and TaAMT1;3a comprise plasma membrane proteins (**Figure 3A**). Furthermore, TaAMT1;1 activities were analyzed by complementation of a yeast strain *Δmep123* in which ${\mathrm{NH}}_{4}^{+}$ uptake is deficient. The results indicated that TaAMT1;1a, TaAMT1;1b, and TaAMT1;3a proteins can transport ${\mathrm{NH}}_{4}^{+}$ in yeast cells (**Figure 3B**). To analyze TaAMT1;1 ${\mathrm{NH}}_{4}^{+}$ transportation function in plants, *TaAMT1;1b* was expressed in the *Arabidopsis qko* mutant in which four *AMT* genes were mutated (Yuan et al., 2007a). Expression of *TaAMT1;1b* driven by the *AtAMT1;1* promoter increased cellular ${\mathrm{NH}}_{4}^{+}$ contents in the mutant, which was similar to the levels in *qko+AtAMT1;1* plants (**Figure 4D**). *qko+TaAMT;1b* was more sensitive to MeA than *qko*, and was slightly more sensitive than the *qko+AtAMT1;1* plants. This sensitivity was exhibited in the relatively shorter roots in comparison to the control (**Figures 4B,C**). These results indicate that TaAMT1;1 transports ${\mathrm{NH}}_{4}^{+}$, and that TaAMT1;1b exhibits similar affinity as AtAMT1;1 against ${\mathrm{NH}}_{4}^{+}$. However, the data showed that MeA-dependent root growth was more severely affected in *qko+TaAMT1;1b* than in *qko+AtAMT1;1* and *qko+TaAMT1;1b* accumulated more ammonium than in *qko+AtAMT1;1*, suggesting that TaAMT1;1b may have higher affinity than AtAMT1;1 in transport of ${\mathrm{NH}}_{4}^{+}$.

The gene expression levels of *AMTs* are sensitive to ${\mathrm{NH}}_{4}^{+}$ in *Arabidopsis* and rice (Sonoda et al., 2003; Loqué et al., 2006). We discovered that *TaAMT1;1a, TaAMT1;1b*, and *TaAMT1;3a* were not altered by ${\mathrm{NH}}_{4}^{+}$ treatment (**Figure 5C**). In addition, the expression of *TaAMT1;1a, TaAMT1;1b, and TaAMT1;3a* genes was induced in the roots and leaves of the wheat plants upon N starvation (**Figure 6C**). As *Arabidopsis* is an ${\mathrm{NH}}_{4}^{+}$-sensitive species, its *AMTs* were suppressed under a high ${\mathrm{NH}}_{4}^{+}$ concentration treatment (Loqué et al., 2006). Conversely, in rice, the ${\mathrm{NH}}_{4}^{+}$-tolerant species *AMT1;1* and *1;2* were highly up-regulated upon supply of ${\mathrm{NH}}_{4}^{+}$ (Sonoda et al., 2003). Suppression of *TaAMT1;3a* in response to ${\mathrm{NH}}_{4}^{+}$ may explain why wheat is also an ${\mathrm{NH}}_{4}^{+}$-sensitive species. The sensitivity to ${\mathrm{NH}}_{4}^{+}$ of barley, which is a close relative of wheat, was explained by the activation of futile ${\mathrm{NH}}_{4}^{+}$ cycling in the membrane (Britto et al., 2001).

Ammonium transporter proteins are involved in a diversity of aspects of plant growth and development. For instance, *Arabidopsis AMT1;3* regulates ${\mathrm{NH}}_{4}^{+}$-triggered lateral branching (Lima et al., 2010). The expression levels of *AMT1;1, AMT1;2, AMT1;3*, and *AMT2;1*, as well as other N metabolic genes, were altered by biotic stresses in *Arabidopsis* (Fagard et al., 2014). Furthermore, *AMT1;1* was involved in *P. syringae-* and *P. cucumerina-*mediated disease in *Arabidopsis* (Pastor et al., 2014). In wheat, expression of three *TaAMTs* was changed by AM fungi infection (Duan et al., 2015), implying a potential regulation of microbe on *TaAMT* regulations. In addition, glutamate supply to the roots induces systemic resistance to rice blast disease in the leaves (Kadotani et al., 2016). These findings suggest that cellular N levels or N signals are closely associated with plant defense. Expression tests of *TaAMT1;1a, TaAMT1;1b, and TaAMT1;3a* in LC and Mini 2761 following urediniospore inoculation of *Pgt* showed that all three genes were induced by the infection only in LC, a wheat stem rust-susceptible line, but not in Mini 2761, a wheat stem rust immune variety. However, the inducement time points were slightly different among the three genes (**Figure 7**). The urediniospore inoculation of *Pgt* in the leaves of LC formed uredinium; however, this was not observed in the leaves of Mini 2761 (**Figure 7**). This implies that *TaAMT1;1a, TaAMT1;1b, and TaAMT1;3a* inducement might require the successful infection of urediniospores into the plant leaves. To understand the mechanism of ${\mathrm{NH}}_{4}^{+}$ on wheat stem rust disease, LC plants were cultured in medium containing either 0 or 1 mM ${\mathrm{NH}}_{4}^{+}$ prior to urediniospore inoculation. The results indicated that the disease class standard levels were lower in the plants grown in the medium without ${\mathrm{NH}}_{4}^{+}$ (**Figure 8**).

Further genetic experiments are required to verify the role of *TaAMT1;1a, TaAMT1;1b*, and *TaAMT1;3a* in wheat stem rust disease, as well as to determine whether the lower disease class standards under N-free conditions are the result of either changes in basal immune response or disruption of N metabolism. The present study characterized the functions of AMTs in wheat plants for the first time. Identification of the interaction between *TaAMTs* and wheat stem rust disease will broaden our understanding of N uptake and metabolism in plant pathogenic infections.

## Author Contributions

TL, BJ, and YX conceived and designed the research. KL, XZ, and XX conducted DNA and RNA isolations. All the experiments were supervised by YX, BJ performed the bioinformatics analysis, and ZW and YG provided figures and tables. YX wrote the manuscript. TL and BJ edited the manuscript. All authors read and approved the manuscript.

## Conflict of Interest Statement

The authors declare that the research was conducted in the absence of any commercial or financial relationships that could be construed as a potential conflict of interest.

## Footnotes

1 http://www.uniprot.org/
