# Supplementary material for: Wheat Ammonium Transporter (AMT) Gene Family: Diversity and Possible Role in Host–Pathogen Interaction with Stem Rust
Source: Front Plant Sci. 2017 Sep 20;8:1637. doi: 10.3389/fpls.2017.01637 (PMC5611643; doi:10.3389/fpls.2017.01637)
Supplement: Supplementary file 2 [file Data_Sheet_2.DOCX]

**Supplementary information**

Fig. S1


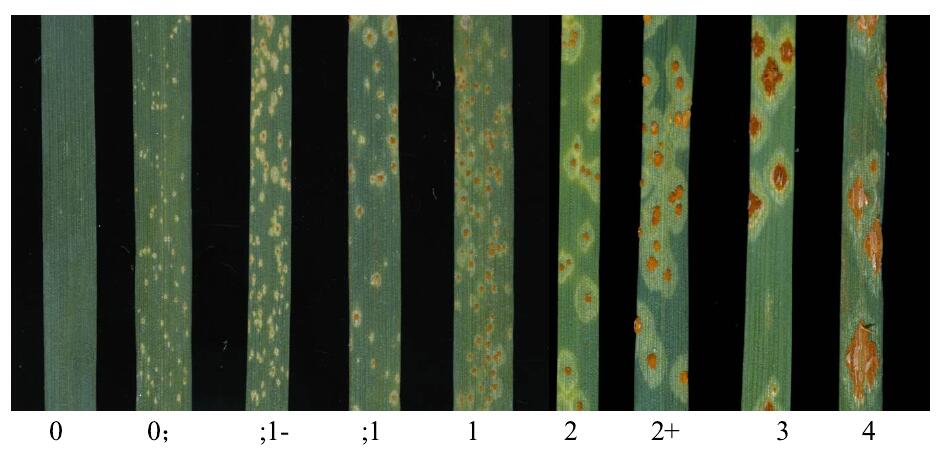


**FIGURE S1** **|** The classification of wheat rust disease symptom. The 2-week-old wheat seedlings were inoculated with [urediniospore](https://www.baidu.com/link?url=ORKCJbNHfrAK7-68-Kb8GzS8LixYAUD2uDHF1bEoc23_DraSzBBDvscjJEWQT2DmST57rFiUeo2qgAdidTc5gC9vI1cop8TKY5llhrs4PLi&wd=&eqid=85d0848b0000a973000000035834ecd4)s of *Pgt*. and 6 classes of standards were photographed.
